# A receptor tyrosine kinase ROR1 inhibitor (KAN0439834) induced significant apoptosis of pancreatic cells which was enhanced by erlotinib and ibrutinib

**DOI:** 10.1371/journal.pone.0198038

**Published:** 2018-06-01

**Authors:** Amir Hossein Daneshmanesh, Mohammad Hojjat-Farsangi, Amineh Ghaderi, Ali Moshfegh, Lotta Hansson, Johan Schultz, Jan Vågberg, Styrbjörn Byström, Elisabeth Olsson, Thomas Olin, Anders Österborg, Håkan Mellstedt

**Affiliations:** 1 Department of Oncology-Pathology, Immune and Gene therapy Lab, Cancer Center Karolinska (CCK), Karolinska University Hospital Solna, Stockholm, Sweden; 2 Karolinska Institutet, Stockholm, Sweden; 3 Department of Hematology, Karolinska University Hospital Solna, Stockholm, Sweden; 4 Kancera AB, Karolinska Institutet Science Park, Stockholm, Sweden; University of South Alabama Mitchell Cancer Institute, UNITED STATES

## Abstract

There is a great unmet medical need in pancreatic carcinoma (PC) for novel drugs with other mechanisms of action than existing. PC cells express the onco-fetal RTK ROR1, absent on most normal post-partem cells. ROR1 is involved in proliferation, survival, EMT and metastasis of tumor cells in various malignancies. A small molecule inhibitor (KAN0439834) (530 Da) targeting the TK domain of ROR1 was developed and the activity in ROR1 expressing human PC cell lines (n = 8) evaluated. The effects were compared to a murine mAb against the external part of ROR1, gemcitabine, erlotinib and ibrutinib. KAN0439834 induced significant apoptosis of the tumor cells. EC_50_ values for KAN0439834 varied between 250–650 nM depending on the cell line. The corresponding values for erlotinib and ibrutinib were 10–40 folds higher. KAN0439834 was much more effective in inducing tumor cell death than the ROR1 mAb although both inhibited ROR1 phosphorylation and downstream non-canonical Wnt pathway molecules. Combination of KAN0439834 with erlotinib or ibrutinib had significant additive effects on tumor cell death. A first-in-class small molecule ROR1 inhibitor (KAN0439834) showed promising in vitro activity against a number of human PC cell lines. Interesting is the additive effects of erlotinib and ibrutinib which warrants further studies as both these agents are in clinical trials for pancreatic carcinoma.

## Introduction

Pancreatic cancer is one of the most aggressive human malignancies and the fourth leading cause of cancer-related death in Europe and the United States [1, 2]. More than 50% of patients with pancreatic cancer are diagnosed with metastases. In 30–40% of patients the disease is localized but surgically not resectable. Even patients with a resectable tumor have a poor outcome. The median survival after surgery including adjuvant therapy is only 2 years [3].

Gemcitabine was for a long time standard first-line treatment of patients with unresectable or metastatic pancreatic cancer. Gemcitabine is still used in adjuvant therapy, while combination regimens for metastatic disease have become the standard ‒ 5-fluorouracil (5-FU)/leucovorin with irinotecan and oxaliplatin (FOLFIRINOX) or nab-paclitaxel with gemcitabine are the most widely used. With these approaches, a progression-free survival (PFS) of 23–31% at 6–7 months has been noted, and a median overall survival (OS) between 8 and 11 months. Thus, there is a great need for innovative medicinal treatments [4].

Receptor tyrosine kinases (RTKs) and associated signaling pathways have important functions in regulating the growth of malignant as well as normal cells. Dysregulation contributes to the growth of malignant cells, self-sufficiency, evasion from apoptosis, unlimited cell replication and metastatic capability [5]. Erlotinib, a tyrosine kinase inhibitor (TKI) of the epidermal growth factor receptor (EGFR), is the only RTK targeting agent, which has been approved for treatment of advanced pancreatic cancer but with minor clinical effect [4]. Ibrutinib, a BTK inhibitor, with off-target effects including EGFR [6] is in phase II-III clinical trials for advanced pancreatic carcinoma (www.clinicaltrials.gov).

ROR1 is a transmembrane protein belonging to the ROR family and one of the twenty RTK families [7], consisting of 937 amino acid residues with extra- and intracellular domains. The extracellular part includes 3 regions: the Ig-like, the cysteine rich (CRD) and the kringle (KNG) domains. The CRD and KNG domains are the ligand binding sites. The intracellular part of ROR1 contains a tyrosine kinase domain, which can be triggered by other intracytoplasmic signaling proteins.

ROR1 is highly expressed during embryogenesis of the central nervous system as well as in the heart, skeletal muscle and pulmonary systems. Adult normal tissues with the exception of adipose tissue, a small early B-cell subset and cells of the upper gastrointestinal tract do not express ROR1 [8]. ROR1 is a highly conserved member of orphan receptors among species and involved in a variety of signaling pathways governing cell growth and epithelial-mesenchymal transition [9]. One of the ligands for ROR1 is Wnt5a [10]. ROR1 has been suggested to be a survival factor for various malignancies including chronic lymphocytic leukemia (CLL), breast cancer, lung adenocarcinoma, ovarian carcinoma, pancreatic carcinoma and glioblastoma [11]. ROR1 seems to play an important role in cell signaling of significance for tumorigenesis as the PI3K/AKT/mTOR pathway and EGFR mediated signaling [9]. Anti-ROR1 monoclonal antibodies (mAbs) and ROR1 specific RNA interference (RNAi) molecules induced apoptosis and growth impairment of several types of malignant cells expressing ROR1 including pancreatic carcinoma [12–14].

In the present study, we analyzed the apoptotic effects of a first-in-class ROR1 small molecule, KAN0439834, targeting the TK domain of ROR1, in a panel of human pancreatic cancer cell lines. The effect of KAN0439834 was compared to gemcitabine, erlotinib, ibrutinib and a murine ROR1 mAb.

## Materials and methods

### Cell lines

The pancreatic cancer cell lines AsPC-1 (ECACC nr. 96020930), BxPC-3 (ECACC nr. 93120816), CFPAC-1 (ATCC nr. CRL-1918), PaCa-2 (ATCC nr. CRL-1420), Capan-2 (ATCC nr. HTB-80), Capan-1(ATCC nr. HTB-79) and HPAF-II (ATCC nr. CRL-1997) (S1 Table) were obtained from European Collection of Authenticated Cell Cultures (ECACC, Porton Down, Salisbury, UK) and American Type Culture Collection (ATCC, Rockville, MD, USA). The pancreatic cell line PaCa-44 was a kind gift from Dr. Matthias Löhr (Karolinska University Hospital Huddinge, Stockholm, Sweden). Capan-1, Capan-2, PaCa-44 and BxPC-3 cells were cultured in DMEM/F12 medium (Gibco, Life Technologies, Karlsruhe, Germany), PaCa-2 cells were cultured in DMEM (high glucose) medium (Gibco) and AsPC-1, CFPAC-1 and HPAF-II cells were cultured in RPMI medium (Gibco) containing 10% FCS (Gibco), 2% glutamine (Biochrom KG, Berlin, Germany) and 100 ug/ml penicillin/ streptomycin (Biochrom KG) at 37°C in a humidified incubator with 5% CO2.

### Enrichment of the ROR1^+^ and ROR1^-^ fractions of the PaCa-2 cell line

Surface ROR1^+^ and ROR1^-^ fractions respectively from the crude PaCa-2 pancreatic cancer cell line (about 60% surface ROR1^+^ cells) were separated by cell sorting using ROR1-APC conjugated antibody (Miltenyi Biotec, Bergisch Gladbach, Germany). FACSAria II Cell Sorter (BD Biosciences, San Jose, CA, USA) was used. The surface ROR1^+^ fraction showed >95% positive cells and in the ROR1 negative fraction <5% ROR1^+^ cells were found (flow-cytometry).

### Anti-ROR1 monoclonal antibodies

Mouse mAbs against ROR1 were generated against the extracellular CRD domain of ROR1 as previously described [15]. One mAb, anti-ROR1 CRD 1D8 (IgG1) was selected for the current study.

### KAN0439834 a small molecule directed against the TK domain of ROR1

KAN0439834 is an oral available small molecule (535 Da) type I tyrosine kinase inhibitor (TKI) targeting the intracellular ROR1 TK domain inhibiting phosphorylation of tyrosine residues 641 and 646. For further details see ref. [16].

### Flow cytometric analysis

Cell surface staining was performed as previously described [15]. Briefly, 10^6^ cells were washed in PBS and suspended in 100 μl of FACS buffer (PBS, 0.1% sodium azide, and 0.5% BSA) and PE conjugated anti-ROR1 (Miltenyi Biotec, Bergisch Gladbach, Germany) mAb and incubated for 20 min at room temperature (RT). Cells were washed with FACS buffer and fixed with 1% paraformaldehyde in PBS. A FACS Canto II flow cytometry (BD Biosciences, San Jose, CA, USA) was used to analyze the frequency of ROR1 expressing cells. 5x10^4^ events were collected and cells were analyzed using the FlowJo software program (Tree Star Inc. Ashland, OR, USA).

### Western blot analysis

10x10^6^ cells were lysed in 200 μl of lysis buffer [0.1% SDS, 1% Triton X-100, 50 mM Tris- HC1, pH 7.4, 150 mM NaCl, 5 mM EDTA, with 1% protease inhibitor cocktail (Sigma-Aldrich, Saint Louis, MO, USA) containing phosphatase inhibitor (Roche, Basel, Switzerland) on ice for 30 min and centrifuged at 13000 rpm. Supernatants were collected and protein concentration measured by the BCA Protein Assay Kit (ThermoFisher Scientific, IL, USA). For Western blot experiment, 10 μg of the cell lysate was run on 10% Bis-Tris SDS-PAGE gel at 120V/90mA for 2 h. After electrophoresis, proteins were transferred to PVDF membrane (Millipore Corporation, Bedford, MA, USA) and blocked overnight at 4°C with 5% BSA in TBS containing 0.1% Tween 20 (TBS-T). Membranes were probed with the respective primary antibodies overnight at 4°C. After washing four times with TBS-T, the filters were incubated with peroxidase-conjugated secondary rabbit anti-goat or mouse antibodies (Dako) for 1 h at room temperature (RT). Membranes were washed four times and developed with the advanced ECL chemoluminescence detection system (GE Healthcare, Uppsala, Sweden). The following antibodies were used to analyze total and phosphorylated (p) proteins; ROR1 (R&D Systems), pROR1 (Tyr 641 and 646, Ser 652), LRP6 and p-LRP6 (Ser 1490), SRC and p-SRC (Tyr 416), PI3K p110δ, AKT and p-AKT (Ser 473), mTOR and p-mTOR (Ser 2448), CREB and p-CREB (Ser 133) (Cell Signaling Technology, Danvers, MA, USA) and p-PI3K p110δ (Santa Cruz Biotechnology, CA, USA).

For the analysis of proteins involved in apoptosis, cleaved poly-(ADP ribose)-polymerase (PARP) and caspase-3 as well as MCL-1 and Bcl-xL were analyzed by Western blot after preparation of cell lysates from the apoptosis experiments (see above). Briefly, 10 μg of protein lysate was run on Western blot gels. Filters were incubated with rabbit anti-PARP, cleaved caspase-3, MCL-1 and Bcl-xL antibodies (Cell Signaling Technology) overnight at 4°C, and subsequently with a secondary peroxidase-conjugated goat anti-rabbit antibody. Blots were developed with the ECL chemiluminescence detection system [15]. For loading control, membranes were probed for β-actin (Sigma-Aldrich). Densitometric quantification was carried out using the ImageJ software (National Institute of Health, USA). Ratios were calculated between the phosphorylated and the total protein.

### Annexin V/PI apoptosis assay

A total of 5x10^4^ cells from the pancreatic cancer cell lines and healthy PBMC were incubated in 24 well plates. After 24 h of incubation, medium was replaced and cells incubated with KAN0439834 (500 nM) in DMEM containing 10% FBS and as controls the anti-ROR1 mAb (10 μg/mL) (optimal dose 10–20 μg/mL in apoptosis experiments [15] and an isotype control mAb (ThermoFisher Scientific). After 24–72 h of incubation at 37°C cells were collected and washed with PBS and suspended in 100 μl of Annexin V binding buffer (BD Biosciences) containing FITC-conjugated Annexin V and PI (Propidium Iodide) (BD Biosciences) and incubated at RT in the dark for 20 min. After incubation, 150 μl of Annexin V binding buffer was added. Viable cells were identified as the double negative Annexin V/PI population. Apoptosis was determined by flow cytometry (FACS Canto II, BD Biosciences) analyzed by the FlowJo software program (Tree Star Inc.).

### Cytotoxicity assay (MTT)

The MTT [3-(4,5-Dimethylthiazol-2-yl)-2,5-Diphenyltetrazolium Bromide] assay (Sigma-Aldrich) was used to measure cytotoxicity. Briefly, 1x10^4^ pancreatic cancer cells were incubated in triplicates in 200 μl of DMEM containing 10% FBS in 96 well plates with KAN0439834 as well as anti-ROR1 mAb, an isotype control mAb, gemcitabine (Eli Lilly, IN, USA), erlotinib (Roche) and ibrutinib (AbbVie, IL, USA). Cells were cultured for 24–72 h. 20 μL MTT solution was added and the cell suspension incubated for 4 h at 37°C. The reaction was stopped by adding MTT solvent (10% SDS in 0.01 M HCL). The plate was read by a microplate reader at 595 nm.

### Human-phospho-RTK array

Phosphorylation of RTKs in PaCa-2 cells was analyzed by the Proteome Profiler Array Kit (Human-phospho-RTK array) (R&D Systems) according to the manufacturer’s protocol. Briefly, membranes were blocked and incubated with 700 μg of PaCa-2 cells lysate overnight at 4°C, washed 3 times in washing buffer and incubated with the secondary antibody (HRP-conjugated anti-phosphotyrosine) for 2 h at RT. Membranes were washed 3 times with washing buffer before development with ECL Western blotting detection reagent (GE Healthcare). Spots of different RTKs were visualized using ECL chemoluminescence detection system (GE Healthcare). The levels of phosphorylated RTKs were densitometrically quantified using ImageJ software (National Institute of Health, USA) and normalized to internal phosphotyrosine-positive controls.

### Proximity ligation assay (PLA)

PLA assay (Duolink In Situ Red Starter Kit Mouse/Rabbit, Sigma-Aldrich) was performed to determine co-localization of ROR1 and LRP6 in PaCa-2 cells. Briefly, untreated and KAN0439834 treated (4 h) PaCa-2 cells were cultured on 8 chamber polystyrene culture slides (Falcon, NY, USA) overnight. Slides were washed twice with PBS and fixed with 4% paraformaldehyde for 20 min and then blocked for 1 h at RT, using blocking buffer. Then, the cells were incubated with mouse anti-human ROR1 (Sigma-Aldrich) and rabbit anti-human LRP6 (Cell Signaling Technology) for overnight at +4^○^C. After washing, cells were incubated with Plus and Minus oligos for 1 h at 37^○^C, followed by ligation and amplification processes for 30 and 100 min, respectively. PLA-signals (red fluorescent spots), each representing a close proximity of these two targets, were examined by Olympus fluorescent microscope and data were captured by an Olympus DP21 camera. The signal obtained by primary irrelevant Abs from mouse and rabbit were used as negative control.

### Statistics

Statistical analyses were done using Student’s t-test and Mann-Whitney U test as appropriate by the GraphPad Prism software (GraphPad Software, Inc., La Jolla, CA, USA). IC50 values were obtained by non-linear curve fitting to the Hills equation using GraphPad Prism of the Accord HTS software (Accelrys Inc., San Diego, CA, USA). P-values < 0.05 were considered significant.

## Results

### ROR1 expression and phosphorylation

**Fig 1:**
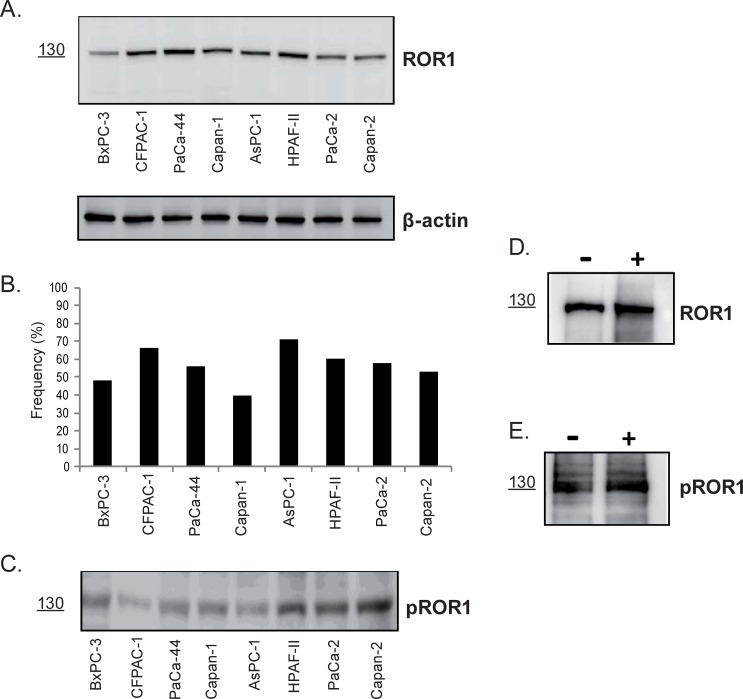
Expression of ROR1 in 8 human pancreatic cancer cell lines. (A) ROR1 expression by Western blot (The fully glycosylated ROR1 protein is 130 kDa) [11]. β-actin was used as loading control. (B) Frequency (mean±SEM) (%) of surface ROR1^+^ cells (flow cytometry). [Statistically significant difference (p-values≤0.01) were: BxPC-3 vs AsPC-1 <0.01; CFPAC-1 vs Capan-1 <0.01; Capan-1 vs AsPC-1 <0.01; AsPC-1 vs Capan-2 = 0.01]. (C) Expression of phosphorylated ROR1 protein (pROR1) (130 kDa). ROR1 and pROR1 expression in surface ROR1 positive (+) and negative (-) PaCa-2 cells (cell sorting). (D) A 130 kDa ROR1 protein was detected (Western blot) in both the ROR1^+^ and ROR1^-^ fractions. (E) ROR1^+^ and ROR1^-^ fractions expressed a pROR1 protein (130 kDa).

Characteristics of the human pancreatic cancer cell lines are shown in S1 Table. All eight cell lines expressed the ROR1 protein as determined by Western blot (Fig 1A) and flow cytometry (Fig 1B). The frequency of surface ROR1^+^ cells varied from 71% (AsPC-1) to 40% (Capan-1). The cell lines expressed phosphorylated ROR1 (pROR1) ‒ a 130 kDa band representing the fully glycosylated ROR1 (Fig 1C). The PaCa-2 cell line was sorted into surface ROR1^+^ and ROR1^-^ fractions respectively. Both these fractions expressed the phosphorylated ROR1 protein (WB) irrespective of whether ROR1 could be detected on the surface or not (Fig 1D and 1E).

### KAN0439834 induced significantly higher rate of apoptosis than anti-ROR1 mAbs

**Fig 2:**
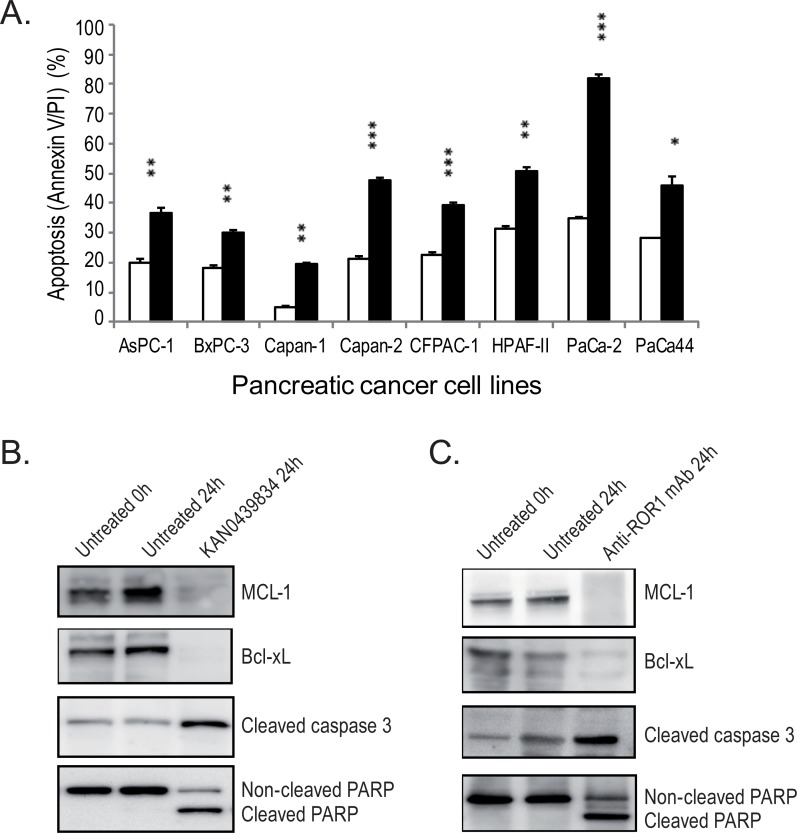
(A) Apoptosis/(Annexin V/PI) (mean±SEM) of KAN0439834 (500 nM) (■) and anti-ROR1 mAb (10 μg/ml) (□) in pancreatic cancer cells after 72 h of incubation. P-values refer to the comparison between anti-ROR1 mAb and KAN0439834 incubated cells. *p = 0.05; **p = 0.01; ***p = 0.001 and ns = not significant. Effects of KAN0439834 (B) and anti-ROR1 mAb (C) on the apoptosis associated proteins MCL-1, Bcl-xL, caspase-3 and PARP proteins after 24 h of incubation. MCL-1 and Bcl-xL proteins were downregulated and caspase-3 and PARP cleaved.

Apoptosis (Annexin V/PI) of the pancreatic cancer cell lines induced by KAN0439834 (500 nM) and the control anti-ROR1 mAb (10 μg/ml) were analyzed. First, a time-kinetics (24–72 h) study was performed using the PaCa-2 cell line. Induction of apoptosis by KAN0439834 was significantly higher than the anti-ROR1 mAb at all time points (p<0.001) (S1 Fig). The optimal time point was 72 h of incubation and used in subsequent experiments. KAN0439834 induced a significantly (p<0.05 ‒ <0.001) higher rate of apoptosis than the anti-ROR1 mAb in all pancreatic cancer cell lines (Fig 2A). PaCa-2 was the most sensitive cell line to KAN0439834 in inducing apoptosis (82%) compared to the anti-ROR1 mAb (35%) (p<0.001). Capan-1 was the most resistant cell line to apoptosis to KAN0439834 (20%) but still more effective than the anti-ROR1 mAb (5%) (p<0.01). KAN0439834 was tested for induction of apoptosis in normal PBMC (B and T cells). No significant killing was observed [16].

Using cell lysates of the PaCa-2 cell line from the 24 h of culture (Fig 2A), it could be shown that KAN0439834 and anti-ROR1 mAb induced both PARP and caspase-3 cleavage as well as downregulation of the MCL-1 and Bcl-xL proteins (Fig 2B and 2C) confirming induction of apoptosis by both compounds.

### Cytotoxicity of KAN0439834 and anti-ROR1 mAb in ROR1 surface positive and negative subpopulations of the PaCa-2 cell line

**Fig 3:**
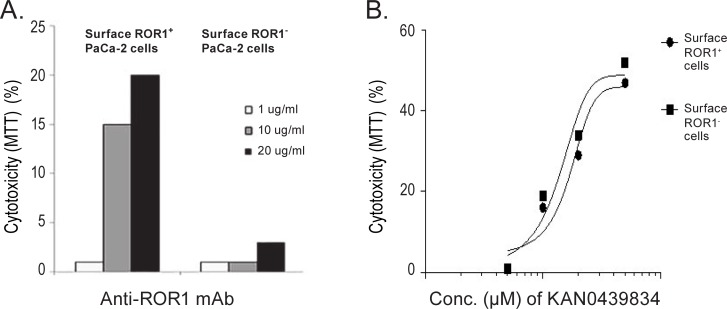
Cytotoxicity (MTT) of anti-ROR1 mAb and KAN0439834 in surface ROR1 positive (+) and negative (-) fractions of PaCa-2 cells. (A) Anti-ROR1 mAb (48 h) (B) KAN0439834 (48 h).

Surface ROR1^+^ and ROR1^-^ fractions of the PaCa-2 cells were obtained by cell sorting. A dose-dependent cytotoxicity (MTT) of the ROR1^+^ PaCa-2 fraction was observed with the ROR1 mAb whereas no killing of the surface negative fraction was observed (Fig 3A). KAN0439834 was equally effective in both surface ROR1^+^ and surface ROR1^-^ subpopulations (Fig 3B). A statistically significant correlation was noted between the frequency of surface ROR1^+^ PaCa-2 fraction and cytotoxicity induced by the anti-ROR1 mAb (p<0.002) (data not shown). No such correlation existed between the frequency of surface ROR1^+^ PaCa-2 fraction and KAN0439834 induced cytotoxicity (data not shown). The data support the notion of expression of ROR1 without a detectable external domain in tumor cells. Similar relations have also been show for EGFR in lung cancer [17].

### Cytotoxicity of KAN0439834, anti-ROR1 mAb, gemcitabine, erlotinib and ibrutinib

There was a close correlation between apoptosis (Annexin V/PI) and cytotoxicity (MTT) in all pancreatic cell lines treated with KAN0439834 in vitro (r = 0.9182; p<0.0001; n = 25) (S2 Fig).

**Fig 4:**
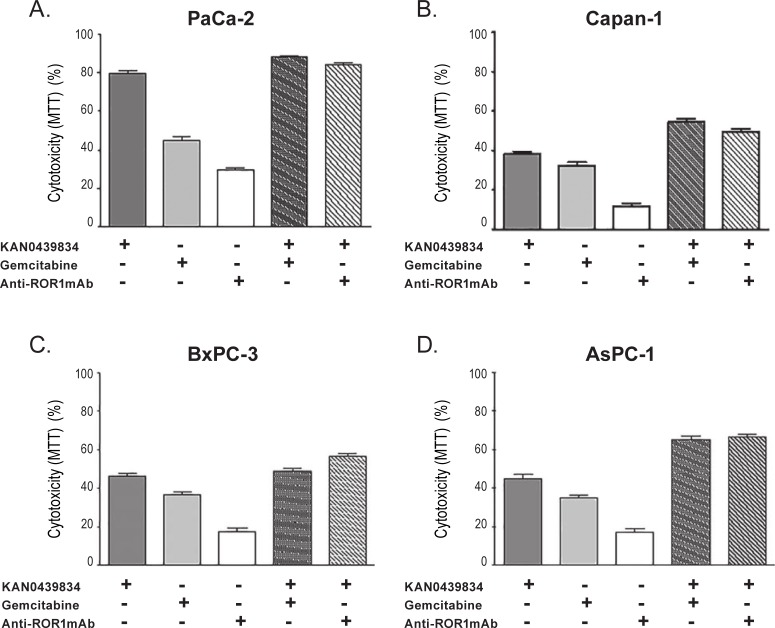
Cytotoxicity (MTT) (mean±SEM) of KAN0439834, gemcitabine and anti-ROR1 mAb in pancreatic cancer cell lines. (A) KAN0439834 in PaCa-2 cell line was significantly more effective compared to gemcitabine (p = 0.0001). KAN0439834 and gemcitabine in combination induced significantly higher cytotoxicity compared to KAN0439834 or gemcitabine alone (KAN0439834 plus gemcitabine vs KAN0439834, p = 0.0033, KAN0439834 plus gemcitabine vs gemcitabine, p = 0.0001). No statistically significant difference comparing KAN0439834 with KAN0439834 plus anti-ROR1 mAb. (B) Cytotoxicity of Capan-1 cells (KAN0439834 vs gemcitabine, p = 0.0483, KAN0439834 plus gemcitabine vs KAN0439834, p = 0.0029, KAN0439834 plus gemcitabine vs gemcitabine, p = 0.0009, KAN0439834 plus anti-ROR1 CRD mAb vs KAN0439834, p = 0.0039, KAN0439834 plus anti-ROR1 mAb vs anti-ROR1 mAb, p = 0.0001) (C) Cytotoxicity of BxPC-3 cells (KAN0439834 vs gemcitabine, p = 0.0044, KAN0439834 plus gemcitabine vs KAN0439834, p = 0.1481, KAN0439834 plus gemcitabine vs gemcitabine, p = 0.0011, KAN0439834 plus anti-ROR1 mAb vs KAN0439834, p = 0.0034, KAN0439834 plus anti-ROR1 mAb vs anti-ROR1 mAb, p = 0.0001). (D) Cytotoxicity of AsPC-1 cells (KAN0439834 vs gemcitabine, p = 0.0106, KAN0439834 plus gemcitabine vs KAN0439834, p = 0.0010, KAN0439834 plus gemcitabine vs gemcitabine, p = 0.0001, KAN0439834 plus anti-ROR1 mAb vs KAN0439834, p = 0.0007, KAN0439834 plus anti-ROR1 mAb vs anti-ROR1 mAb, p = 0.0001).

Cytotoxicity (MTT) of KAN0439834, anti-ROR1 mAb, gemcitabine, erlotinib and ibrutinib alone and in combinations were compared. KAN0439834 in combination with gemcitabine induced a statistically significant higher cytotoxicity in PaCa-2 cells than KAN0439834 alone (p = 0.0033) but the difference (88% vs 80%) was small (Fig 4A). Adding the ROR1 mAb to KAN0439834 did not augment cytotoxicity (ns). Similar results were obtained with the Capan-1 cell line with the exception that combining KAN0439834 and the anti-ROR1 mAb increased the efficacy compared to KAN0439834 alone (p = 0.0039) (Fig 4B). In BxPC-3 cells, the addition of ROR1 mAb, but not gemcitabine, enhanced cytotoxicity of KAN0439834 (p<0.003 and p = 0.14, respectively) (Fig 4C). The highest additive cytotoxic effect was observed in AsPC-1 cells, both when gemcitabine (p = 0.001) or the ROR1 mAb (p = 0007) was added to KAN0439834 (Fig 4D). Information on the four other cell lines (CFPAC-1, Capan-2, PaCa-44 and HPAF-II) using KAN0439834 and gemcitabine are presented in S3A–S3D Fig. Data were similar to those described above.

**Fig 5:**
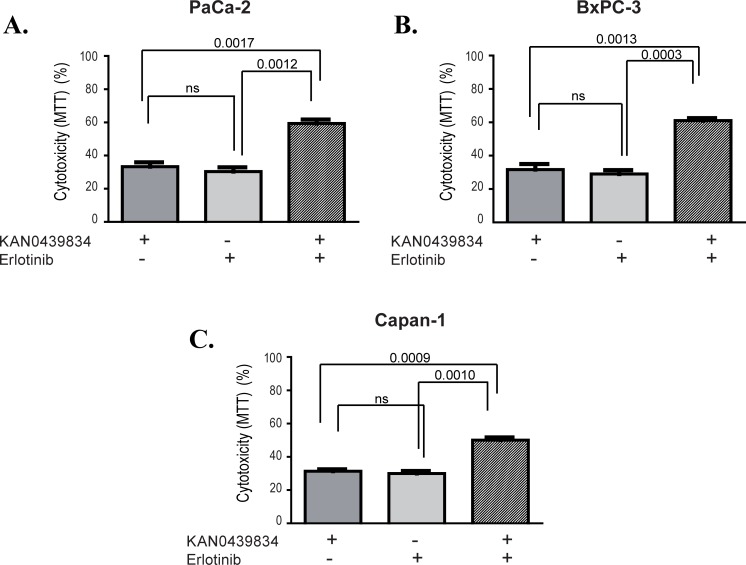
Cytotoxicity (%) (mean±SEM) of three pancreatic cell lines cultured (72 h) with KAN0439834 and erlotinib alone and in combination using EC_30_ value concentrations for the different drugs and cell lines respectively. Statistics are shown at the top.

**Fig 6:**
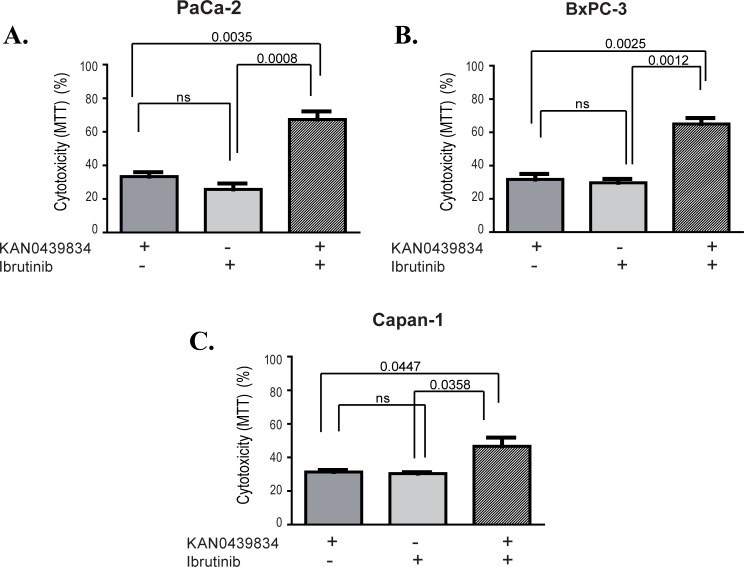
Cytotoxicity (%) (mean±SEM) of three pancreatic cell lines cultured (72 h) with KAN0439834 and ibrutinib alone and in combination using EC_30_ value concentrations for the different drugs and cell lines respectively. Statistics are shown at the top.

Cytotoxic effects of KAN0439834 and erlotinib alone and in combination was studied in PaCa-2, BxPC-3 and Capan-1 pancreatic cancer cell lines (Fig 5A–5C). EC_50_ values were 10–20 times higher for erlotinib as compared to KAN0439834 (Table 1). Combination of KAN0439834 and erlotinib showed significant additive effects in all three cell lines compared to either drug alone. Similarly, KAN0439834 and ibrutinib were compared in three cell lines. EC_50_ values for ibrutinib was 20–40 times higher as compared to KAN0439834 (Table 1). The combination of these two drugs showed significant additive effects (Fig 6A–6C).

**Table 1:** EC50 values (nM) for KAN0439834, erlotinib and ibrutinib in three pancreatic cancer cell lines.

Cell line	KAN0439834 EC_50_ (nM)	Erlotinib EC_50_ (nM)	Ibrutinib EC_50_ (nM)
**PaCa-2**	**250**	**> 5000**	**> 10000**
**BxPC-3**	**500**	**> 5000**	**> 15000**
**Capan-1**	**650**	**> 10000**	**> 15000**

### KAN0439834 inhibited phosphorylation of ROR1

**Fig 7:**
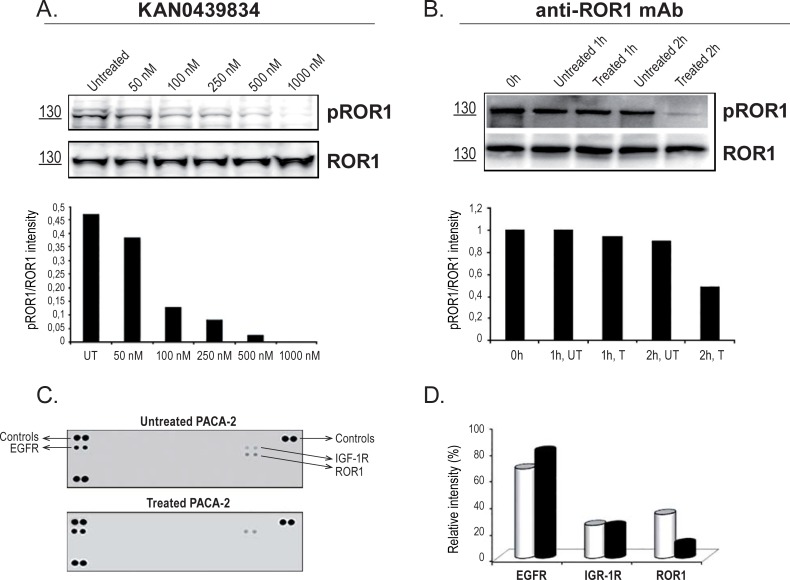
KAN0439834 and anti-ROR1 mAb inhibited ROR1 phosphorylation in pancreatic carcinoma cells (PaCa-2). Total ROR1 (ROR1) and phosphorylated ROR1 (pROR1) after 2 h incubation with KAN439834 (A) and (B) anti-ROR1 mAb. Intensity values of pROR1 relative to total ROR1 are shown. Effects on phosphorylation of RTKs (Phospho-RTK array) in PaCa-2 cells by KAN0439834. (C) PaCa-2 cells were treated with 500 nM of KAN0439834 for 2 h ((■) or untreated (UT) for 2 h (□)). EGFR, ROR1 and IGF-1R were detected in this specific array. Inhibition of ROR1 phosphorylation could only be noted after exposure to KAN0439834. (D) Relative intensity values of pEGFR, pIGF-1R and pROR1 to total EGFR, IGF-1R and ROR1.

KAN0439834 induced a dose-dependent inhibition of ROR1 phosphorylation which could be detected at 50 nM and was complete at 1000 nM (IC50 for inhibition of ROR1 phosphorylation was <100 nM) (Fig 7A). In contrast, at the optimal cytotoxic dose of the anti-ROR1 mAb (10 μg/mL) only a 50% decrease in phosphorylation of ROR1 was noted (Fig 7B). Inhibition of ROR1 phosphorylation by KAN0439834 was confirmed by a human-phospho-RTK array using percolated nitrocellulose membranes containing 48 different human-phospho-RTKs (Fig 7C and 7D). The assay also showed that KAN0439834 did not dephosphorylate EGFR and IGF-1R, kinases which are overexpressed in pancreatic carcinoma cells [18, 19]. Gemcitabine, erlotinib and ibrutinib did not dephosphorylate ROR1 (data not shown).

### Effects of KAN0439834 on ROR1 associated signaling molecules

**Fig 8:**
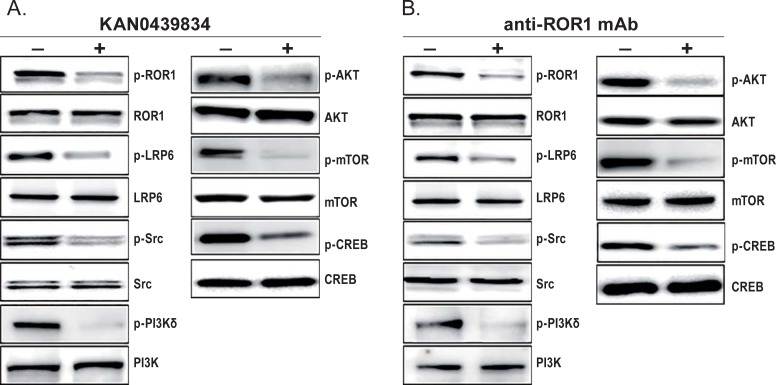
Effects of KAN0439834 and anti-ROR1 mAb on ROR1 associated signaling molecules in the pancreatic cancer cell line PaCa-2. Western blots showed inhibition of ROR1, LRP6, SRC, PI3Kδ, AKT, mTOR and CREB phosphorylation after incubation (2 h) with (A) KAN0439834 (500 nM) and (B) anti-ROR1 mAb (10 μg/ml). (+) treated cells and (-) untreated cells.

PaCa-2 cells were incubated with KAN0439834 or anti-ROR1 mAb and molecules associated with ROR1 signaling were analyzed (Fig 8A and 8B). Both KAN0439834 and anti-ROR1 mAb dephosphorylated LRP6 (a co-receptor for ROR1), SRC, PI3K, AKT, mTOR as well as the transcription factor CREB.

### ROR1/LRP6 heterodimerization and effects of KAN0439834

ROR1 and the co-receptor LRP6 were physically bound to each other (heterodimerized) on PaCa-2 cells (PLA assay). No dissociation was seen after incubation with KAN0439834 (S4A and S4B Fig), but inhibition of ROR1 phosphorylation as well as LRP6 was observed (Fig 8A).

## Discussion

Pancreatic carcinoma is characterized by mutations and/or silencing of tumor suppressor genes, as TP53 and Smad4, mutation of KRAS and overexpression of receptor tyrosine kinases (RTKs) such as EFGR, VEGFR, IGF-1R, and RON. In addition, ROR1 was shown to be expressed in pancreatic cancer with no expression in normal pancreatic tissues [8].

ROR1 plays a key role in normal embryogenesis. It is absent in most mature tissues, but highly expressed in CLL and other hematological malignancies as well as in solid tumors as breast, ovarian, lung and pancreatic cancers [11, 20]. High level of ROR1 expression is associated with a more aggressive and poor prognosis disease [11, 21]. ROR1 regulates the expression of the genes involved in epithelial-mesenchymal transition as well as sustains a favorable survival balance between the pro-survival PI3K-AKT and the pro-apoptotic p38 signaling in lung cancer [22, 23]. Knockdown of ROR1 induced apoptosis of tumor cells [12, 24]. Wnt5a, a ligand for ROR1 and ROR2, induced ROR1/ROR2 hetero-oligomerization and enhanced leukemia chemotaxis and proliferation [25]. Binding of Wnt5a to ROR1 promoted cancer cell survival and metastasis [10]. ROR1 may dimerize with the co-receptor LRP6 upon ligand activation transmitting downstream signals by SRC and the Wnt-canonical and non-canonical pathways [26].

A first-in-class small molecule KAN0439834, active against ROR1 was selected as a lead candidate following an extensive drug development process including an HTS campaign against a recombinant intracellular part of ROR1 and subsequent optimization by phenotypic screening using CLL cells. Here, the antitumor activity of KAN0439834 was explored in vitro using a panel of eight human pancreatic carcinoma cell lines expressing the phosphorylated full-length ROR1 and EGFR.

The main finding was that KAN0439834 had a higher killing efficacy than gemcitabine, erlotinib, ibrutinib and an anti-ROR1 mAb respectively as single agents. Additive cytotoxic effects were seen by gemcitabine, erlotinib and ibrutinib. The higher cytotoxic effects of KAN0439834 compared to anti-ROR1 mAb might be due to off-target effects of the small molecule as binding to other kinases at high concentrations (10 μM) could be noted[16]. Furthermore, complete blocking of ROR1 mediated signaling (dephosphorylation of the TK domain) could not be achieved by the mAb which was the case for the small molecule. In addition to inhibition of ROR1, LRP6 and SRC phosphorylation, KAN0439834 also inhibited phosphorylation of the PI3K/AKT/mTOR pathway, which is important in tumorigenesis [27].

The combination of KAN0439834 and anti-ROR1 mAb, two drugs targeting different structures of the ROR1 molecules, enhanced cytotoxicity in about 25% of the tested cell lines. This is in line with reports showing that the combination of anti-HER2 mAb and lapatinib might increase the apoptotic effects of HER2 expressing breast cancer cells as compared to either drug alone but not consistently [28, 29].

Previous studies using flow cytometry have shown two distinct clones of the PaCa-2 cell line [30]. As expected, the anti-ROR1 mAb induced apoptosis of the surface ROR1^+^ PaCa-2 cells but not of surface ROR1^-^ cells, while KAN0439834 induced apoptosis of both fractions. ROR1 has multiple N-glycosylation sites that generate post-translationally modified ROR1 regulating ROR1 localization. The N-glycosylation sites are necessary for trafficking of ROR1 to the membrane and the function of ROR1 [31]. It is possible that differences in glycosylation and trafficking of ROR1 might affect the surface expression of ROR1 in cancer cells.

Gemcitabine has been shown to possess different cytotoxic capability in pancreatic cell lines. PaCa-2 cells were the most sensitive, Capan-1 the most resistant, and BxPC-3 cells had an intermediate sensitivity [32, 33], a similar profile as for KAN0439834. In all tested cell lines, KAN0439834 induced a higher cytotoxicity than gemcitabine. Cell lines expressing the wild-type KRAS have been shown to be more sensitive to erlotinib (EGFR inhibitor) than those with mutated KRAS [34]. However, KAN0439834 was equally effective in inducing apoptosis of pancreatic cancer cell lines irrespective of KRAS mutation.

Erlotinib and ibrutinib have been shown to induce cell death of lung and pancreatic cancer cell lines expressing members of the EGFR receptor family [35, 36]. Both drugs dephosphorylated EGFR [35]. The killing efficacy of these two drugs and KAN0439834 was compared using suboptimal concentrations (EC_30_ values). Erlotinib and ibrutinib had a similar killing capacity as KAN0439834 at EC_30_ and a clear additive effect when they were combined with KAN0439834. KAN0439834 inhibited phosphorylation of ROR1 but not EGFR and BTK, while erlotinib and ibrutinib dephosphorylated EGFR [35, 36] but not ROR1 [16] suggesting different mechanisms of action. The additive effect of KAN0439834, erlotinib and ibrutinib might partly be explained by that all three compounds cross-talk with the EGFR pathway [17, 35, 36] and furthermore, both KAN0439834 and ibrutinib are inhibitors of Wnt pathways [11, 36].

EGFR may heterodimerize with HER2 activating the tyrosine kinase domain of EGFR [37]. Erlotinib had no effect on heterodimerization of EGFR and HER2 in breast cancer cells but the complex proteins (EGFR and HER2) were dephosphorylated [38]. Similarly, KAN0439834 did not affect the ROR1 and LRP6 complex (heterodimerization) but inhibited phosphorylation of the complex components.

KAN0439834 inhibited phosphorylation of ROR1, LRP6 and SRC as well as molecules of the non-canonical pathway which preceded apoptosis similar to what have been reported in CLL cells [26, 39]. SRC proteins binding to the cytoplasmic part of RTKs have been shown to be activated in lung, breast and pancreatic carcinoma cells [40]. ROR1 phosphorylation leads to interaction with and phosphorylation of SRC which is suggested to be an important step in tumorigenesis [23]. KAN0439834 also downregulated the PI3K/AKT/mTOR pathway. Activated ROR1 has been shown to trigger AKT in lung adenocarcinoma cells and ROR1 knockdown induced dephosphorylation of AKT and apoptosis. Moreover, ROR1 has been shown to utilize kinase dependent and independent mechanisms to sustain a favorable balance between PI3K/AKT pro-survival signals and the pro-apoptotic p38 pathway [23]. A significant association between the expression of ROR1 and activated AKT/CREB enhancing tumor cell growth has also been reported [14]. Up-regulation of the PI3K/AKT pathway correlated with an impaired gemcitabine-induced apoptosis in pancreatic cancer cells [41]. All these signaling molecules were downregulated by KAN0439834 which might contribute to tumor cell induced apoptosis.

In summary, a first-in-class small molecule active against phosphorylated ROR1, KAN0439834, was studied for killing capacity of pancreatic carcinoma cell lines. Tumor cell induced apoptosis by KAN0439834 was enhanced by erlotinib and ibrutinib as well as by gemcitabine. The findings are of potential therapeutic interest as the three targeting agents have different but complementary mechanisms of action in inducing tumor cell death and ibrutinib additionally also on the tumor microenvironment [42]. Further studies are warranted of this novel therapeutic principle in pancreatic carcinoma as well as in other ROR1 expressing tumors alone and specifically in combination with other targeting drugs or standard of care.

## Supporting information

S1 Fig Time-kinetics for cytotoxicity (MTT) (mean±SEM) of the PaCa-2 cell line incubated with KAN0439834 and anti-ROR1 mAb in vitro. (DOC)

S2 Fig Correlation between apoptosis (Annexin V/PI) and cytotoxicity (MTT) of the different pancreatic cell lines incubated with KAN0439834 for 72 h (r = 0.9182; p < 0.0001; n = 25). (DOC)

S3 Fig Cytotoxicity (MTT) (mean±SEM) of KAN0439834, anti-ROR1 mAb and gemcitabine alone and in combinations. (A) CFPAC-1 cell line (KAN0439834 vs gemcitabine, p = 0.0029, KAN0439834 plus gemcitabine vs KAN0439834, p = 0.0033, KAN0439834 plus gemcitabine vs gemcitabine, p = 0.0001). (B) Capan-2 cell line (KAN0439834 vs gemcitabine, p = 0.0018, KAN0439834 plus gemcitabine vs KAN0439834, p = 0.0030, KAN0439834 plus gemcitabine vs gemcitabine, p = 0.0002). (C) HPAF-II cell line (KAN0439834 vs gemcitabine, p = 0.005, KAN0439834 plus gemcitabine vs KAN0439834, p = 0.0285, KAN0439834 plus gemcitabine vs gemcitabine, p = 0.0003). (D) PaCa-44 cell line (KAN0439834 vs gemcitabine, p = 0.0004, KAN0439834 plus gemcitabine vs KAN0439834, p = 0.0059, KAN0439834 plus gemcitabine vs gemcitabine, p = 0.0001).
(DOC)

S4 Fig Heterodimerization of ROR1 and LRP6 shown by proximity ligation assay (PLA). (A) In situ PLA showing co-localization of ROR1 with LRP6 molecules in untreated PaCa-2 cells (63 X). Each red spot represents a close proximity of ROR1 and LRP6 molecules inside or on the surface of PaCa-2 cells. (B) In situ PLA assay showing co-localization of ROR1 with LRP6 molecules in PaCa-2 cells (63 X) after treatment with KAN0439834 (1 μM) (4 h). Each red spot represents a close proximity of ROR1 and LRP6 molecules inside or on the surface of PaCa-2 cells.
(DOC)

S1 Table Characteristics of the human pancreatic cancer cell lines. (DOC)
