# Supplementary material for: A receptor tyrosine kinase ROR1 inhibitor (KAN0439834) induced significant apoptosis of pancreatic cells which was enhanced by erlotinib and ibrutinib
Source: PLoS One. 2018 Jun 1;13(6):e0198038. doi: 10.1371/journal.pone.0198038 (PMC5983484; doi:10.1371/journal.pone.0198038)
Supplement: S1 Fig — (DOC) [file pone.0198038.s001.doc]

**Supplementary Figure S1**

Time-kinetics for cytotoxicity (MTT) (mean±SEM) of the PaCa-2 cell line incubated with KAN0439834 and anti-ROR1 mAb in vitro.
