# Supplementary material for: A receptor tyrosine kinase ROR1 inhibitor (KAN0439834) induced significant apoptosis of pancreatic cells which was enhanced by erlotinib and ibrutinib
Source: PLoS One. 2018 Jun 1;13(6):e0198038. doi: 10.1371/journal.pone.0198038 (PMC5983484; doi:10.1371/journal.pone.0198038)
Supplement: S2 Fig — (DOC) [file pone.0198038.s002.doc]

**Supplementary Figure S2**

Correlation between apoptosis (Annexin V/PI) and cytotoxicity (MTT) of the different pancreatic cell lines incubated with KAN0439834 for 72 h (r = 0.9182; p < 0.0001; n = 25).
