# Supplementary material for: A receptor tyrosine kinase ROR1 inhibitor (KAN0439834) induced significant apoptosis of pancreatic cells which was enhanced by erlotinib and ibrutinib
Source: PLoS One. 2018 Jun 1;13(6):e0198038. doi: 10.1371/journal.pone.0198038 (PMC5983484; doi:10.1371/journal.pone.0198038)
Supplement: S3 Fig — (A) CFPAC-1 cell line (KAN0439834 vs gemcitabine, p = 0.0029, KAN0439834 plus gemcitabine vs KAN0439834, p = 0.0033, KAN0439834 plus gemcitabine vs gemcitabine, p = 0.0001). (B) Capan-2 cell line (KAN0439834 vs gemcitabine, p = 0.0018, KAN0439834 plus gemcitabine vs KAN0439834, p = 0.0030, KAN0439834 plus gemcitabine vs gemcitabine, p = 0.0002). (C) HPAF-II cell line (KAN0439834 vs gemcitabine, p = 0.005, KAN0439834 plus gemcitabine vs KAN0439834, p = 0.0285, KAN0439834 plus gemcitabine vs gemcitabine, p = 0.0003). (D) PaCa-44 cell line (KAN0439834 vs gemcitabine, p = 0.0004, KAN0439834 plus gemcitabine vs KAN0439834, p = 0.0059, KAN0439834 plus gemcitabine vs gemcitabine, p = 0.0001). (DOC) [file pone.0198038.s003.doc]

**Supplementary Figure S3**

**Cytotoxicity (MTT) (mean±SEM) of KAN0439834, anti-ROR1 mAb and gemcitabine alone and in combinations.** (**A**) CFPAC-1 cell line (KAN0439834 vs gemcitabine, p=0.0029, KAN0439834 plus gemcitabine vs KAN0439834, p=0.0033, KAN0439834 plus gemcitabine vs gemcitabine, p=0.0001). (**B**) Capan-2 cell line (KAN0439834 vs gemcitabine, p=0.0018, KAN0439834 plus gemcitabine vs KAN0439834, p=0.0030, KAN0439834 plus gemcitabine vs gemcitabine, p=0.0002). (**C**) HPAF-II cell line (KAN0439834 vs gemcitabine, p=0.005, KAN0439834 plus gemcitabine vs KAN0439834, p=0.0285, KAN0439834 plus gemcitabine vs gemcitabine, p=0.0003). (**D**) PaCa-44 cell line (KAN0439834 vs gemcitabine, p=0.0004, KAN0439834 plus gemcitabine vs KAN0439834, p=0.0059, KAN0439834 plus gemcitabine vs gemcitabine, p=0.0001).
