# Supplementary material for: A receptor tyrosine kinase ROR1 inhibitor (KAN0439834) induced significant apoptosis of pancreatic cells which was enhanced by erlotinib and ibrutinib
Source: PLoS One. 2018 Jun 1;13(6):e0198038. doi: 10.1371/journal.pone.0198038 (PMC5983484; doi:10.1371/journal.pone.0198038)
Supplement: S4 Fig — (A) In situ PLA showing co-localization of ROR1 with LRP6 molecules in untreated PaCa-2 cells (63 X). Each red spot represents a close proximity of ROR1 and LRP6 molecules inside or on the surface of PaCa-2 cells. (B) In situ PLA assay showing co-localization of ROR1 with LRP6 molecules in PaCa-2 cells (63 X) after treatment with KAN0439834 (1 μM) (4 h). Each red spot represents a close proximity of ROR1 and LRP6 molecules inside or on the surface of PaCa-2 cells. (DOC) [file pone.0198038.s004.doc]

**Supplementary Figure S4**

**Heterodimerization of ROR1 and LRP6 shown by proximity ligation assay (PLA).** (**A**) In situ PLA showing co-localization of ROR1 with LRP6 molecules in untreated PaCa-2 cells (63 X). Each red spot represents a close proximity of ROR1 and LRP6 molecules inside or on the surface of PaCa-2 cells. (**B**) In situ PLA assay showing co-localization of ROR1 with LRP6 molecules in PaCa-2 cells (63 X) after treatment with KAN0439834 (1 µM) (4 h). Each red spot represents a close proximity of ROR1 and LRP6 molecules inside or on the surface of PaCa-2 cells.
