# Supplementary material for: A receptor tyrosine kinase ROR1 inhibitor (KAN0439834) induced significant apoptosis of pancreatic cells which was enhanced by erlotinib and ibrutinib
Source: PLoS One. 2018 Jun 1;13(6):e0198038. doi: 10.1371/journal.pone.0198038 (PMC5983484; doi:10.1371/journal.pone.0198038)
Supplement: S1 Table — (DOC) [file pone.0198038.s005.doc]

**Supplementary Table 1.** Characteristics of the human pancreatic cancer cell lines *

| **Cell lines** | **Origin of cell line** | **Tumor cell type** | **Tumor cell differentiation** | **Genotype** |
| --- | --- | --- | --- | --- |
| AsPC-1 | Ascites | Adenocarcinoma | Poor | KRAS, P53, P16, SMAD |
| BxPC-3 | Primary tumor | Adenocarcinoma | Moderate to poor | KRAS, P53, P16, SMAD |
| Capan-1 | Liver metastasis | Adenocarcinoma | Well | KRAS, P53, P16, SMAD |
| Capan-2 | Primary tumor | Adenocarcinoma | Well | KRAS, P53, P16, SMAD |
| CFPAC-1 | Liver metastasis | Adenocarcinoma | Well | KRAS, P53, P16, SMAD |
| HPAF-II | Ascites | Adenocarcinoma | Well | KRAS, P53, P16, SMAD |
| PaCa-2 | Primary tumor | Adenocarcinoma | Poor | KRAS, P53, P16, SMAD |
| PaCa-44 | Primary tumor | Adenocarcinoma | Poor | KRAS, P53, P16, SMAD |

* All cell lines expressed EGFR
